# Association between the Phytochemical Index and Lower Prevalence of Obesity/Abdominal Obesity in Korean Adults

**DOI:** 10.3390/nu12082312

**Published:** 2020-07-31

**Authors:** Jihyun Im, Minkyeong Kim, Kyong Park

**Affiliations:** 1 Department of Food and Nutrition, Yeungnam University, 280 Daehak-ro, Gyeongsan, Gyeongbuk 38541, Korea; jhim@ynu.ac.kr; 2 Division of Brain Diseases, Center for Biomedical Sciences, Korea National Institute of Health, 187 Osongsaengmyeong2-ro, Osong-eup, Heungdeok-gu, Cheongju-si, Chungcheongbuk-do 28159, Korea; kminkyeong94@gmail.com

**Keywords:** phytochemical, obesity, abdominal obesity, women, South Korea

## Abstract

The relatively high levels of vegetable consumption have highlighted the need to examine the association between phytochemical intake and disease prevention. We examined the association between the phytochemical index (PI) and obesity/abdominal obesity among Korean adults. We analyzed the data of 57,940 adults aged ≥ 19 years obtained from the Korea National Health and Nutrition Examination Survey. We calculated PI using the 24 h recall data, and multivariable-adjusted odds ratios (ORs) and 95% confidence intervals (CIs) were estimated using logistic regression models. Dose–response patterns were analyzed using restricted cubic spline regression. After multivariable adjustment, a higher PI was found to be associated with a lower prevalence of obesity and abdominal obesity; this association was notable in women (obesity, OR: 0.86, CI: 0.78–0.94, *p* for trend = 0.01; abdominal obesity, OR: 0.81, CI: 0.73–0.90, *p* for trend < 0.001). Spline regression showed linearity of the associations between PI and obesity/abdominal obesity in women. Our findings suggested that maintaining a phytochemical-rich diet may help to prevent obesity and abdominal obesity, especially in women, as an increased PI corresponded to lower prevalence of obesity. This study, using evidence-based data, highlighted the importance of consuming plant-derived foods to prevent obesity.

## 1. Introduction

Obesity and abdominal obesity are risk factors for chronic diseases, with steadily increasing rates reported among Korean adults [1,2]. Of particular concern is the fact that the obesity rates are expected to increase at a faster rate in Korea than in other member countries belonging to the Organization for Economic Cooperation and Development [3]. Weight gain can increase the risk of chronic diseases (e.g., diabetes mellitus and heart disease [4,5]) and premature mortality [6].

While various nutritional epidemiological studies have been conducted to identify key dietary factors affecting the risk of obesity, consuming a diet rich in vegetables, fruits, and whole grains has been shown to be effective for weight control [7,8,9]. Phytochemicals are physiologically active substances commonly found in plant-based foods, especially fruits and vegetables, that have evident antioxidant and anti-obesity-related functions [10]. A phytochemical index (PI) has previously been proposed to evaluate the disease prevention effect of phytochemicals in epidemiological studies [11,12,13,14,15,16]. A cross-sectional study of 356 children aged 7–10 years in Iran showed a negative association between phytochemical intake and obesity [12]. The Tehran Lipid and Glucose study involving 2567 adults reported that the participants in the highest PI quartile had a 66% lower risk of abdominal obesity than those in the lowest quartile [16]. However, few studies have been conducted to determine the association between phytochemical intake and obesity in Asian populations. Particularly, Korean individuals have a relatively high vegetables consumption compared to individuals from other parts of the world [17]. Additionally, it is unclear whether there is a dose–response relationship between phytochemicals and obesity/abdominal obesity, even in individuals who consume more vegetables. Therefore, it is pertinent to determine whether a linear relationship exists between phytochemical intake and obesity among Korean adults.

This study aimed to investigate the association between PI and obesity/abdominal obesity and determine dose–response patterns for the associations of the same among Korean men and women using the 2008–2018 data from the Korea National Health and Nutrition Examination Survey (KNHANES).

## 2. Materials and Methods

### 2.1. Data Source and Study Population

The KNHANES is a large-scale cross-sectional study that was initiated in 1998. It is conducted to identify health-related consciousness and behavior, health levels, and food and nutrition status among South Koreans. It consists of a health examination, health interview, and nutritional survey. In this study, we used the survey data of 11 years (2008–2018), and information concerning physical activity, an important confounding factor of the research hypothesis, was collected.

In total, 93,028 people participated in the KNHANES 2008–2018. Of these, participants with the following characteristics were excluded from the analysis: (1) those aged <19 years (n = 20,935); (2) those with missing sampling weight information (n = 11,950); (3) those with extremely low or high total daily energy intake (<500 kcal or >5000 kcal, respectively) (n = 1039); (4) women who were pregnant or lactating (n = 822); (5) those who had missing data or variables required to calculate PI, body mass index (BMI) values, or waist circumference (WC) (n = 342). Finally, 57,940 participants were included in our study.

Written informed consent was obtained from all the study participants to use and analyze their data, and this study was approved by the Institutional Review Board of the Korea Centers for Disease Control and Prevention (Approval numbers: 2008-04EXP-01-C, 2009-01CON-03-2C, 2010-02CON-21-C, 2011-02CON-06-C, 2012-01EXP-01-2C, 2013-07CON-03-4C, 2013-12EXP-03-5C, and 2018-01-03-P-A).

### 2.2. Demographic and Lifestyle Data

Among the data collected using the health interview survey, demographic variables, such as age, sex, education level, and household income, were used for analysis, as were data concerning smoking status, alcohol consumption, and physical activity. The participants were classified into five categories, based on their age, as follows: 19–29, 30–39, 40–49, 50–59, and ≥60 years. Moreover, the participants were classified according to their education level (lower than elementary school, middle school, high school, and university graduation or higher) and, then, subdivided into two categories, namely, (i) lower than high school education and (ii) high school educated or higher. The household income levels were analyzed in terms of sex and age in accordance with the monthly household equivalence income, and variables were reclassified through applying the quartile of each group into two categories, namely, (i) mid-low or lower and (ii) mid-high or higher-income levels. Regarding smoking status, the participants were divided into current smokers and non-smokers. Alcohol consumption was calculated based on the alcohol consumption frequency within the previous 12 months and the participants were classified as (i) non-drinkers and (ii) active alcohol drinkers (according to whether they had consumed alcohol within this timeframe).

Regarding physical activity, the amount of time the participants exercised per week during the period from 2008 to 2013 was calculated using data for the number of days/time spent walking or undertaking moderate/intense physical activity and through multiplying the exercise intensity. From 2014 to 2018, the survey method for determining physical activity was further subdivided into work and leisure; hence, the average values in relation to work and leisure in terms of physical activity were used [18]. Metabolic equivalents (METs) were calculated as the physical activity levels per week (METs-h/week) and presented as tertiles according to the distribution.

### 2.3. Anthropometric Measurement and Diagnosis of Obesity

Trained staff conducted the health examination in the survey, including anthropometric measurements, in accordance with a standardized protocol [19]. Height and weight were measured using a standardized height and weight scale, with participants standing upright on the scale after removing their shoes and socks while wearing an examination gown [ 19]. A tape measure was used to measure the WC at a midpoint between the lowest part of a participant’s last rib and the top of the iliac ridge [19]. BMI was calculated by dividing the participants’ body weight (kg) by height squared (m^2^).

In general, Asians, including those lower BMIs, have a higher risk of cardiometabolic diseases. In addition, the association between BMI, body fat percentage, and body fat distribution differ between Asian and other populations; therefore, specific obesity cut-off points for Asian and Pacific populations have been established [20]. According to the World Health Organization criteria for Asian populations, obesity is classified as follows: BMI < 23 kg/m^2^, underweight and normal; BMI 23 to < 25 kg/m^2^, overweight; and BMI ≥ 25 kg/m^2^, obese [20]. Abdominal obesity has been defined as WC ≥ 90 and ≥ 85 cm for men and women, respectively, according to the Korean Society for the Study of Obesity criteria [21].

### 2.4. Nutritional Survey Data and PI

The KNHANES used a 24 h recall survey to collect information on food consumption one day before the survey [22]. The trained staff conducted face-to-face interviews. Supplementary materials were used to collect specific data on the survey items and enhance the recall skills [22]. This study calculated PI, which was based on the daily nutrient intake data, using the 24 h recall data present in the KNHANES.

PI is generally defined as the percentage of the calories received through the intake of phytochemical-rich foods divided by the total number of calories consumed during the day [11]. We used a modified PI that had been previously developed based on a Korean diet [23]. Briefly, the food groups included in the PI calculation were whole grains, vegetables, fruits, legumes, soybeans and soybean products, nuts and seeds, and olive oil, as proposed by McCarty [11]. As seaweed is often consumed by Koreans [24], it was also included in the PI calculations. Further detailed information on the PI calculation has previously been described [23].

### 2.5. Statistical Analysis

We conducted a statistical analysis considering the stratification variables, the primary sampling unit, and through reflecting the proper weighting of survey segments of the KNHANES. The participants’ characteristics are presented as frequencies and percentages for categorical variables. To analyze the association between PI and obesity/abdominal obesity, multivariable logistic regression analyses were used to calculate the odds ratios (ORs) and 95% confidence intervals (CIs). The *P*-value for the trend in the PI quintiles using linear regression analysis was evaluated using the median value of the category, as a continuous variable. Multiple confounding factors were identified based on the preliminary analysis and a literature review [12,25,26]. To systematically account for potential confounding factors, three covariate models were evaluated, as follows: Model 1 was unadjusted; Model 2 was adjusted for age; and Model 3 was further adjusted for the education level, household income, smoking status, alcohol consumption, physical activity, total energy intake, and level of intake of meat and meat products, sweets, and dairy products. Spline regression allows the regression to have a different slope above and below a certain point, and the function itself is continuous, although non-parametric. In this study, the potential linearity was examined by modeling a continuous exposure variable (PI) using restricted cubic spline regression with three Knots based on Akaike’s information criterion, which balances flexibility versus overfitting [26]. All covariates in model 3 were adjusted in the spline regression. Statistical Analysis System version 9.4 software (SAS Institute, Cary, NC, USA) was used to perform all analyses, and the statistical significance level for all tests was set at α = 0.05.

## 3. Results

### 3.1. General Participant Characteristics According to PI

The general characteristics of the participants are shown in Table 1. The median PI for quintiles 1, 2, 3, 4, and 5 were 2.32, 6.60, 11.40, 17.90, and 30.85, respectively. The average age of the participants tended to decrease with increasing PI levels, and the percentage of elderly participants ≥60 years were 29.22%, 29.10%, 35.17%, 39.77%, and 44.38% in the 1st, 2nd, 3rd, 4th, and 5th PI quintiles, respectively. The proportion of participants who had mid-high or higher household income was 43.38%, 48.05%, 50.52%, 53.71%, and 55.32% in the 1st, 2nd, 3rd, 4th, and 5th PI quintiles, respectively. Approximately >50% of the participants had graduated from high school or were educated to a higher level. Approximately 30% of the participants were obese (BMI ≥ 25), and the proportion of this ranged from 31.99% to 33.15% in the PI quintiles. Smoking and alcohol consumption tended to be negatively associated with PI levels. The proportion of participants who were active smokers and consumed alcohol was 27.76%, 23.43%, 17.66%, 13.21%, and 9.13%, and 76.29%, 75.86%, 71.77%, 67.33%, and 61.03% in the 1st, 2nd, 3rd, 4th, and 5th PI quintiles, respectively.

### 3.2. Association between PI and Obesity/Abdominal Obesity

Table 2 shows the ORs for obesity and abdominal obesity according to the PI for Korean adults (men and women). Compared with the lowest PI quintile in all models, except for Model 1, women in the highest PI quintile showed a significantly lower prevalence of obesity (Model 3, OR: 0.86, 95% CI: 0.78–0.94, *p* for trend = 0.01). In contrast, there was no statistically significant association between PI and the prevalence of obesity among men. The association between PI and abdominal obesity showed a similar pattern. In fully adjusted models, we found a significantly lower prevalence of abdominal obesity in women with the highest PI quintile than in those with the lowest (Model 3, OR: 0.81, 95% CI: 0.73–0.90, *p* for trend < 0.001). In men, the significant association was observed between PI and abdominal obesity for the specific intake quintile. A higher PI was associated with a lower prevalence of abdominal obesity (Model 3, *p* for trend = 0.03); this association was evident for the 4th quintile (Model 3, OR: 0.89, 95% CI: 0.79–0.99) compared to the lowest quintile.

### 3.3. Dose–Response Association between PI and Obesity/Abdominal Obesity

**Figure 1:**
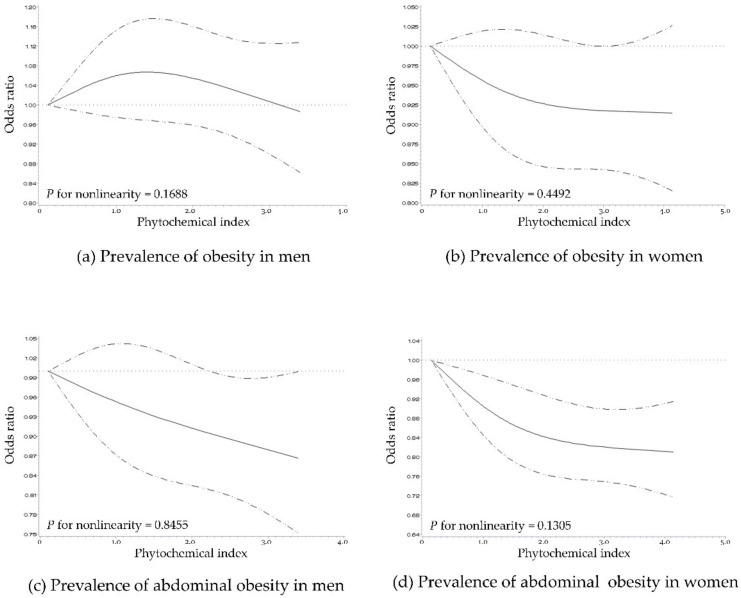
Multivariable adjusted odds ratios (95% confidence intervals) for the nonlinear relationship between phytochemical index and prevalence of obesity and abdominal obesity in Korean men and women. The prevalences of obesity in men (a) and women (b) and the prevalence of abdominal obesity in men (c) and women (d) were evaluated using restricted cubic splines. The model was adjusted for age (19–29, 30–39, 40–49, 50–59, and ≥60 years), education level (lower than high school education and high school educated or higher), household income (mid-low or lower and mid-high or higher), smoking status (active smokers and non-smokers), alcohol consumption (drinkers and non-drinkers), physical activity (low, mid, and high), total energy intake (continuous), and level of intake of meat and meat products (continuous), sweets (continuous), and dairy products (continuous). Obesity was defined as having a body mass index ≥25 kg/m^2^ according to the World Health Organization criteria for Asian populations. Abdominal obesity was defined as having a waist circumference ≥90 cm and ≥85 cm for men and women, respectively, according to the Korean Society for the Study of Obesity criteria.

Figure 1 shows the spline curve for the association between PI and the prevalence of obesity and abdominal obesity in the form of dose–response relationships. A higher PI showed an inverse linear association with the prevalence of abdominal obesity in men and women. In addition, the inverse linear association between PI and obesity was seen only in women (all *p* for nonlinearity > 0.05).

## 4. Discussion

Our study aimed to investigate the association between PI and obesity/abdominal obesity among Korean men and women since the vegetable intake in this population is relatively higher compared to other populations in general. Overall, women tended to have a higher PI than men. Women with a higher PI had a lower prevalence of obesity and abdominal obesity. In addition, the dose–response relationship appeared generally linear or monotonic at the PI ranges seen in this population, showing lower prevalence of obesity and abdominal obesity at higher PI levels. However, among men, no association was observed between PI and the prevalence of obesity, although the association between PI and abdominal obesity seemed to be negatively linear.

Our results showed that individuals, and especially women, who consume a significant amount of phytochemical-rich foods have a reduced prevalence of obesity and abdominal obesity, in line with the corresponding results from previous studies [25,27,28]. Indeed, one study using the KNHANES 2008–2011 data for Korean adults reported that a higher intake of total flavonoids resulted in lower prevalence of obesity (18%) and abdominal obesity (19%); however, in men, this association was not confirmed [25]. Additionally, a study using the KNHANES 2007–2016 data for Korean women found that the prevalence of obesity was approximately 11% lower in women with the highest intake of lycopene than in those with the lowest (OR: 0.89, CI: 0.83–0.96). Moreover, a significant negative correlation was identified between the intake of α-carotene, total carotenoids, vitamin A, γ-tocopherols, and lycopene and abdominal obesity (all *p* for trend < 0.05) [25]. Furthermore, an epidemiological study of 864 adults aged ≥39 years in Japan also found an association between the serum carotenoid levels and abdominal obesity in women, with no corresponding significant association found in men [28].

The following mechanisms may explain the positive effects of phytochemical-rich vegetable intake on obesity and abdominal obesity. Among the phytochemical types, polyphenols are the most effective at regulating fat metabolism through stimulating lipolysis and inducing fatty acid oxidation through multistep reactions in the mitochondria and peroxisomes [29,30]. Further, they have been shown to inhibit the proliferation of adipocytes and induce apoptosis [16]. Moreover, a recent study reported that anthocyanin increases the AMP-activated protein kinase (AMPK) (a key molecule in the regulation of fatty acid oxidation pathways) levels and lowers the carnitine palmitoyltransferase-1 levels [31]. Resveratrol has been proposed as an anti-obesity agent that increases fatty acid oxidation through upregulation of AMPK and PGC-1α [32,33] and downregulation of peroxisome proliferator-activated receptor gamma and CCAAT-enhancer-binding protein [34]. Hydroxycitric acid inhibits fat production by reducing acetyl-CoA and using it as a precursor for fatty acid and cholesterol biosynthesis [13]. Furthermore, flavonoids, such as quercetin, naringenin, genistein, kaempferol, hesperidin, and rutin, are effective for preventing obesity, specifically through reducing fat accumulation in the body [10].

Generally, visceral fat accumulation has been reported to be higher in men than in women, probably because premenopausal women can accumulate more body fat than men of the same age before reaching the visceral adipose tissue amounts found in men [35]. The observed association of a higher PI with a lower prevalence of obesity and abdominal obesity in women, but not in men, could be attributed to the interaction among the phytochemical intake levels and the complex effects of sex hormones [36,37,38,39,40]. In this study, the phytochemical intake was higher among women than among men, which could be attributed to the fact that many women in our study consumed phytochemicals to gain the positive health benefit afforded by phytochemicals. Moreover, certain types of phytochemicals, such as resveratrol and isoflavones, have similar structures to estrogens, and they are known to imitate or influence the role of estrogen in the body [10,36,39]. Specifically, isoflavones have been considered as a substitute for hormone therapy and can be consumed as a source of phytochemical-rich foods [36]. Given that phytoestrogen intake can have a positive effect on diseases caused by the lack of estrogen [36,37], it is likely that the intake of phytochemical-rich foods may have a complex effect on obesity prevalence by assisting the women’s bodies in controlling hormones. Further research should be conducted to clarify the cause-and-effect relationship of the role of phytochemicals on obesity, considering the sex differences.

Although a growing body of evidence has shown that a diet rich in vegetables, whole grains, legumes, and nuts (foods rich in phytochemicals) has beneficial effects on the prevention of obesity and other chronic disease, little is known regarding the optimal intake levels of phytochemicals from epidemiological studies. Therefore, in this work, we highlighted the potential beneficial effects of phytochemicals in the community setting. In addition, there may be some misunderstanding that occasional fruit and vegetable consumption or some portions of these foods in a diet may be sufficient to manage or prevent obesity. We showed that the higher intake of phytochemicals resulted in a well-controlled body weight, showing that there is a dose–response relationship. Further, these results were found even among individuals known to consume high amounts of vegetables. Our study provided scientific evidence regarding the importance of plant-rich diets and those including plentiful phytochemicals. Finally, we showed that the beneficial effects of a phytochemical-rich diet were more pronounced in women.

This study had several limitations and strengths. First, the PI calculation was conducted using a 24 h recall data, and this is not representative of habitual dietary intake measurement; therefore, potential misclassifications may have occurred. However, a trained investigator obtained the dietary information using standardized protocols, and participants who showed extreme levels of energy consumption were excluded to minimize the likelihood of error. Second, the KNHANES is a cross-sectional study and, owing to the research design, it was difficult to establish a clear causal relationship between PI and the risk of obesity and abdominal obesity. Third, the BMI values cannot be used to distinguish between fat tissue and lean muscle mass; therefore, the BMI results may be particularly misleading in participants who are elderly, pregnant, or have muscular physiques. A WC measurement may not define where the fat is stored within the body (subcutaneous or visceral) and is not indicative of abdominal obesity. Finally, PI was calculated based on the calories in terms of the food consumed; therefore, food items containing no or very few calories were not included in the PI calculation. Nevertheless, as this study used the KNHANES, which is a nationally representative sample of the Korean population, our results can be generalized to Korean adults. Moreover, this evidence-based study highlighted the importance of including plant-derived foods in the dietary guidelines to prevent or manage obesity.

## 5. Conclusions

In conclusion, increased consumption of phytochemical-rich foods was found to be beneficial for obesity and abdominal obesity, with associated linearity even in a population already consuming an increased amount of vegetables. Of note, this benefit was more pronounced in women than in men. Future large-scale prospective cohort or clinical studies using dietary data with fewer measurement errors (such as 3 day recalls or longer) should be conducted in the Korean population to confirm the clear preventative effects of phytochemicals on obesity and abdominal obesity.

## Figures and Tables

**Table 1:** General participant characteristics according to the phytochemical index quintile, KNHANES 2008–2018 (n = 57,940).

	Phytochemical Index Quintiles				
	Q1	Q2	Q3	Q4	Q5
**Number of Participants**	11,588	11,588	11,588	11,588	11,588
**Phytochemical Index, Median (range)**	2.32 (0.00–4.46)	6.60 (4.46–8.86)	11.40 (8.86–14.31)	17.90 (14.31–22.74)	30.85 (22.74–98.91)
**Sex**					
Men	5801 (50.06)	5412 (46.70)	4913 (42.40)	4260 (36.76)	3515 (30.33)
Women	5787 (49.94)	6176 (53.30)	6675 (57.60)	7328 (63.24)	8073 (69.67)
**Age (years)**					
19–29	2279 (19.67)	1527 (13.18)	1141 (9.85)	839 (7.24)	583 (5.03)
30–39	2372 (20.47)	2452 (21.16)	1966 (16.97)	1528 (13.19)	1125 (9.71)
40–49	1984 (17.12)	2339 (20.18)	2251 (19.43)	2186 (18.86)	1848 (15.95)
50–59	1567 (13.52)	1898 (16.38)	2154 (18.59)	2427 (20.94)	2889 (24.93)
≥60	3386 (29.22)	3372 (29.10)	4076 (35.17)	4608 (39.77)	5143 (44.38)
**Household Income**					
Mid-low or lower	6499 (56.62)	5960 (51.95)	5677 (49.48)	5313 (46.29)	5129 (44.68)
Mid-high or higher	4980 (43.38)	5513 (48.05)	5797 (50.52)	6164 (53.71)	6351 (55.32)
**Education Level**					
Lower than high school education	3597 (32.54)	3678 (33.17)	4148 (37.39)	4394 (39.72)	4681 (42.16)
High school educated or higher	7456 (67.46)	7410 (66.83)	6947 (62.61)	6669 (60.28)	6421 (57.84)
**Smoking Status**					
Non-smoker	8185 (72.24)	8644 (76.57)	9277 (82.34)	9753 (86.79)	10,242 (90.87)
Current smoker	3146 (27.76)	2645 (23.43)	1990 (17.66)	1485 (13.21)	1029 (9.13)
**Alcohol Consumption**					
No	2685 (23.71)	2723 (24.14)	3178 (28.23)	3668 (32.67)	4387 (38.97)
Yes	8640 (76.29)	8557 (75.86)	8078 (71.77)	7561 (67.33)	6869 (61.03)
**Body Mass Index (kg/m^2^)**					
<23	5259 (45.38)	5153 (44.47)	5098 (43.99)	5065 (43.71)	4866 (41.99)
23–25	2487 (21.46)	2636 (22.75)	2709 (23.38)	2816 (24.30)	2894 (24.97)
≥25	3842 (33.15)	3799 (32.78)	3781 (32.63)	3707 (31.99)	3828 (33.03)
**Physical Activity ^1^**					
Low	4229 (38.21)	3673 (33.09)	3555 (32.02)	3432 (31.00)	3237 (29.12)
Mid	3723 (33.64)	3735 (33.65)	3798 (34.21)	3736 (33.75)	3817 (34.34)
High	3115 (28.15)	3692 (33.26)	3748 (33.76)	3902 (35.25)	4062 (36.54)

KNHANES, Korea National Health and Nutrition Examination Survey; Q, quintile. Values are presented as n (%). ^1^ Physical activity level was calculated as metabolic equivalent task-hours per week (METs-h/week) and categorized in tertiles.

**Table 2:** Odds ratios (95% confidence intervals) for obesity and abdominal obesity according to the phytochemical index quintile.

	Phytochemical Index Quintiles					*p* for Trend
	Q1	Q2	Q3	Q4	Q5	
**Obesity ^1^**						
**Men**						
N	4780	4780	4781	4780	4780	
No. of cases (%)	1708 (35.73)	1847 (38.64)	1771 (37.04)	1731 (36.21)	1744 (36.49)	
Model 1	1	1.14 (1.03–1.26)	1.06 (0.96–1.17)	1.02 (0.92–1.13)	1.04 (0.94–1.15)	0.73
Model 2	1	1.10 (1.00–1.22)	1.04 (0.94–1.15)	1.02 (0.92–1.14)	1.07 (0.96–1.19)	0.64
Model 3	1	1.09 (0.99–1.21)	1.05 (0.95–1.17)	1.03 (0.92–1.14)	1.05 (0.94–1.17)	0.85
**Women**						
N	6807	6808	6808	6808	6808	
No. of cases (%)	2010 (29.53)	1929 (28.33)	1989 (29.22)	2075 (30.48)	2153 (31.62)	
Model 1	1	0.95 (0.87–1.03)	1.01 (0.92–1.10)	1.06 (0.97–1.16)	1.15 (1.05–1.26)	<0.001
Model 2	1	0.86 (0.79–0.94)	0.85 (0.77–0.93)	0.84 (0.77–0.92)	0.83 (0.76–0.91)	0.001
Model 3	1	0.87 (0.79–0.95)	0.87 (0.79–0.96)	0.87 (0.79–0.96)	0.86 (0.78–0.94)	0.01
**Abdominal Obesity ^2^**						
**Men**						
N	4780	4780	4781	4780	4780	
No. of cases (%)	1377 (28.81)	1393 (29.14)	1366 (28.57)	1388 (29.04)	1408 (29.46)	
Model 1	1	1.04 (0.94–1.16)	0.98 (0.88–1.10)	0.99 (0.89–1.10)	1.03 (0.93–1.15)	0.81
Model 2	1	1.00 (0.90–1.10)	0.91 (0.82–1.02)	0.88 (0.79–0.98)	0.90 (0.81–1.00)	0.01
Model 3	1	0.99 (0.89–1.10)	0.93 (0.83–1.04)	0.89 (0.79–0.99)	0.90 (0.81–1.01)	0.03
**Women**						
N	6807	6808	6808	6808	6808	
No. of cases (%)	1783 (26.19)	1730 (25.41)	1758 (25.82)	1836 (26.97)	1926 (28.29)	
Model 1	1	1.00 (0.91–1.09)	1.04 (0.95–1.14)	1.09 (0.99–1.19)	1.20 (1.09–1.32)	<0.001
Model 2	1	0.88 (0.80–0.97)	0.82 (0.74–0.91)	0.79 (0.72–0.87)	0.77 (0.70–0.86)	<0.001
Model 3	1	0.89 (0.80–0.98)	0.84 (0.76–0.93)	0.84 (0.76–0.93)	0.81 (0.73–0.90)	<0.001

Q, quintile. Model 1, Unadjusted; Model 2, Adjusted for age (19–29, 30–39, 40–49, 50–59, and ≥60 years); Model 3, Model 2 plus additional adjustment for the education level (lower than high school education and high school educated or higher), household income (mid-low or lower and mid-high or higher), smoking status (non-smokers), alcohol consumption (drinkers and non-drinkers), physical activity (low, mid, and high), and intake levels of total energy (continuous), meat and meat products (continuous), sweets (continuous), and dairy products (continuous). ^1^ Obesity was defined as having a body mass index ≥25 kg/m^2^ according to the World Health Organization criteria for Asian populations. ^2^ Abdominal obesity was defined as having a waist circumference ≥90 cm and ≥85 cm for men and women, respectively, according to the Korean Society for the Study of Obesity criteria.

## References

[1] Korea Centers for Disease Control and Prevention (2020). Korea Health Statistics 2018: Korea National Health and Nutrition Examination Survey (KNHANES Ⅶ-3).

[2] Korean Society for the Study of Obesity. Diagnosis of Obesity. http://general.kosso.or.kr/html/?pmode=obesityDiagnosis.

[3] Sassi F., Devaux M., Cecchini M., Rusticelli E. (2009). The Obesity Epidemic: Analysis of Past and Projected Future Trend in Selected OECD Countries.

[4] Park C. (1997). Obesity and cardiovascular disease. Korean Circ. J..

[5] Korean Society for the Study of Obesity. Comorbidities of Obesity. http://general.kosso.or.kr/html/?pmode=obesityDisease.

[6] Di Angelantonio E., Bhupathiraju S.N., Wormser D., Gao P., Kaptoge S., de Gonzalez A.B., Cairns B.J., Huxley R., Jackson C.L., Joshy G. (2016). Body-mass index and all-cause mortality: Individual-participant-data meta-analysis of 239 prospective studies in four continents. Lancet.

[7] He K., Hu F.B., Colditz G.A., Manson J.E., Willett W.C., Liu S. (2004). Changes in intake of fruits and vegetables in relation to risk of obesity and weight gain among middle-aged women. Int. J. Obes. Relat. Metab. Disord. J. Int. Assoc. Study Obes..

[8] Liu S., Willett W.C., Manson J.E., Hu F.B., Rosner B., Colditz G. (2003). Relation between changes in intakes of dietary fiber and grain products and changes in weight and development of obesity among middle-aged women. Am. Soc. Clin. Nutr..

[9] Serdula M.K., Byers T., Mokdad A.H., Simoes E., Mendlein J.M., Coates R.J. (1996). The Association between Fruit and Vegetable Intake and Chronic Disease Risk Factors. Epidemiology.

[10] Holubková A., Penesová A., Šturdík E., Mošovská S., Mikušová L. (2012). Phytochemicals with potential effects in metabolic syndrome prevention and therapy. Acta Chim. Slovaca.

[11] McCarty M.F. (2004). Proposal for a dietary “phytochemical index”. Med. Hypotheses.

[12] Eslami O., Khoshgoo M., Shidfar F. (2020). Dietary phytochemical index and overweight/obesity in children: A cross-sectional study. BMC Res. Notes.

[13] Rupasinghe H.P., Sekhon-Loodu S., Mantso T., Panayiotidis M.I. (2016). Phytochemicals in regulating fatty acid beta-oxidation: Potential underlying mechanisms and their involvement in obesity and weight loss. Pharmacol. Ther..

[14] Vincent H.K., Bourguignon C.M., Taylor A.G. (2010). Relationship of the dietary phytochemical index to weight gain, oxidative stress and inflammation in overweight young adults. J. Hum. Nutr. Diet..

[15] Carnauba R.A., Chaves D.F., Baptistella A.B., Paschoal V., Naves A., Buehler A.M. (2016). Association between high consumption of phytochemical-rich foods and anthropometric measures: A systematic review. Int. J. Food Sci. Nutr..

[16] Bahadoran Z., Golzarand M., Mirmiran P., Saadati N., Azizi F. (2013). The association of dietary phytochemical index and cardiometabolic risk factors in adults: Tehran Lipid and Glucose Study. J. Hum. Nutr. Diet..

[17] OECD Indicators (2017). Health at A Glance 2017.

[18] Ainsworth B.E., Haskell W.L., Leon A.S., Jacobs D.R., Montoye H.J., Sallis J.F., Paffenbarger R.S. (1993). Compendium of Physical Activities: Classification of energy costs of human physical activities. Med. Sci. Sports Exerc..

[19] Korea Centers for Disease Control and Prevention (2016). Guidelines for Examination and Inspection (2016–2018).

[20] World Health Organization The Asia Pacific Perspective: Redefining Obesity and Its Treatment. www.wpro.who.int/nutrition/documents/.../Redefiningobesity.pdf.

[21] Korean Society for the Study of Obesity. Diagnosis and Evaluation of Obesity. http://general.kosso.or.kr/html/?pmode=BBBS0001300003&page=2&smode=view&seq=86&searchValue=&searchTitle=strTitle&setRowCount=undefined.

[22] Korea Centers for Disease Control and Prevention, Ministry of Health and Welfare (2016). Guidelines for Nutrition Investigation (2015).

[23] Kim M., Park K. (2020). Association between phytochemical index and metabolic syndrome. Nutr. Res. Pract..

[24] Han M.R., Ju D.L., Park Y.J., Paik H.Y., Song Y. (2015). An Iodine Database for Common Korean Foods and the Association between Iodine Intake and Thyroid Disease in Korean Adults. Int. J. Thyroidol..

[25] Ham D., Kim S., Jun S., Kang M., Joung H. (2018). Association between antioxidant vitamin intake and obesity among Korean women: Using the Korea National Health and Nutrition Examination Survey 2007 ~ 2016. J. Nutr. Health.

[26] Harrell F.E. (2015). Regression Modeling Strategies: With Applications to Linear Models, Logistic and Ordinal Regression, and Survival Analysis.

[27] Kim S.A., Kim J., Jun S., Wie G.A., Shin S., Joung H. (2020). Association between dietary flavonoid intake and obesity among adults in Korea. Appl. Physiol. Nutr. Metab..

[28] Suzuki K., Inoue T., Hioki R., Ochiai J., Kusuhara Y., Ichino N., Osakabe K., Hamajima N., Ito Y. (2006). Association of abdominal obesity with decreased serum levels of carotenoids in a healthy Japanese population. Clin. Nutr..

[29] Feillet-Coudray C., Sutra T., Fouret G., Ramos J., Wrutniak-Cabello C., Cabello G., Cristol J.P., Coudray C. (2009). Oxidative stress in rats fed a high-fat high-sucrose diet and preventive effect of polyphenols: Involvement of mitochondrial and NAD(P)H oxidase systems. Free Radic. Biol. Med..

[30] SHimoda H., Tanaka J., Kikuchi M., Fukuda T., Ito H., Hatano T., Yoshida T. (2009). Effect of polyphenol-rich extract from walnut on diet-induced hypertriglyceridemia in mice via enhancement of fatty acid oxidation in the liver. J. Agric. Food Chem..

[31] Wu T., Tang Q., Gao Z., Yu Z., Song H., Zheng X., Chen W. (2013). Blueberry and Mulberry Juice Prevent Obesity Development in C57BL/6 Mice. PLoS ONE.

[32] Rivera L., Moron R., Zarzuelo A., Galisteo M. (2009). Long-term resveratrol administration reduces metabolic disturbances and lowers blood pressure in obese Zucker rats. Biochem. Pharmacol..

[33] Lagouge M., Argmann C., Gerhart-Hines Z., Meziane H., Lerin C., Daussin F., Messadeq N., Milne J., Lambert P., Elliott P. (2006). Resveratrol improves mitochondrial function and protects against metabolic disease by activating SIRT1 and PGC-1alpha. Cell.

[34] Aguirre L., Fernandez-Quintela A., Arias N., Portillo M.P. (2014). Resveratrol: Anti-obesity mechanisms of action. Molecules.

[35] Lemieux S., Prud’homme D., Bouchard C., Tremblay A., Després J.P. (1993). Sex differences in the relation of visceral adipose tissue accumulation to total body fatness. Am. J. Clin. Nutr..

[36] Lee J., Heo J., Park Y., Park H. (2012). Survey on the Consumption of the Phytoestrogen Isoflavone in Postmenopausal Korean Women. J. Korean Soc. Menopause.

[37] Kim M., Choi M., Sung C. (2004). The Study of Pytoestrogen Intake and Bone Mineral Density of Vegetarian and Nonvegetarian Postmenopausal Women. Korean J. Commun. Nutr..

[38] Kim B.-J. (2010). Obesity and Sex Hormones. J. Korean Soc. Study Obes..

[39] Choi H.M. (2017). Nutrition in the 21st Century.

[40] Son S.M., Im H.S., Kim J.H., Lee J.H., Seo J.S., Son J.M. (2018). Clinical Nutrition.

